# Working memory and the need for explainable AI – Scenarios from healthcare, social media and insurance

**DOI:** 10.1016/j.heliyon.2025.e41871

**Published:** 2025-01-10

**Authors:** M. Liebherr, E. Gößwein, C. Kannen, A. Babiker, S. Al-Shakhsi, V. Staab, B.J. Li, R. Ali, C. Montag

**Affiliations:** a Department of General Psychology: Cognition, University Duisburg-Essen, Duisburg, Germany; b Department of Mechatronics, University Duisburg-Essen, Duisburg, Germany; c Department of Molecular Psychology, Institute of Psychology and Education, Ulm University, Ulm, Germany; d College of Science and Engineering, Hamad Bin Khalifa University, Doha, Qatar; e Wee Kim Wee School of Communication and Information, Nanyang Technological University, Singapore

**Keywords:** Cognition, Working memory, XAI, Intellect, Personality, Context

## Abstract

Explainable AI (XAI) is discussed as an important feature of AI systems that is required for professional, ethical and commercial reasons. XAI is particularly needed in AI systems that remain relatively closed, i.e. black box, on how to make decisions whether for complexity or commercial sensitivity. Still, many factors play an important role on whether people perceive a need for XAI. The personal relevance of the AI system might play a role, but also individual differences in personality traits or cognitive functions. In order to test these assumptions, 215 participants (129 female) underwent an online experiment testing their objective working memory capacity and completed the Intellect facet of the Big Five Personality traits (as a proxy for self-assessed cognitive capacity). Further, all participants were presented three AI systems scenarios of relatively different degrees of (personal) relevance and, hence, personal investment: skin cancer detection (i), the personalization of social media feeds (ii), and the amount to pay for a car insurance (iii). We observed that people reported a higher need for XAI in scenarios where they were more personally invested (health, payment for insurance > social media); for signficant differences between these scenarios see the result section. Interestingly, working memory capacity was not associated with XAI needs across the three domains, but self-reported Intellect showed a mild positive association with need for an AI explanation in the context of the insurance story. The present study represents a first attempt to investigate objective working memory capacity in the context of need for XAI. Beyond that, the study indicates that personal investment is crucial for determining the need for XAI.

## 1 Introduction

In recent years, the rapid expansion of sophisticated artificial intelligence (AI) systems, especially those deployed in critical decision-making contexts, has raised concerns regarding transparency and accountability. To tackle these apprehensions, eXplainable AI (XAI) has emerged as a promising approach to enhance the transparency of AI systems. For example, Google's DeepMind could be mentioned. Here, an AI system for detecting eye diseases was developed, where the system not only diagnoses conditions from retinal scans but also provides detailed explanations and visualizations of how it arrived at its conclusions. By providing human-understandable explanations for AI decisions, XAI fosters positive attitudes toward AI, as summarized in the theoretical framework of the IMPACT model [1]. The success of XAI in enhancing trust relies not only on the quality of explanations but also on technological factors, the specific application context, and also the nuanced interaction of users and their cognitive processes. Our present study focus is on the relevance of working memory capacity in interactions with XAI, alongside examining the contextual dynamics shaping its necessity.

Working memory refers to a cognitive system with limited capacity that is responsible for temporarily holding and manipulating information necessary for complex tasks such as reasoning, decision-making, and problem-solving [2]. The most widely accepted framework conceptualizes working memory as a system comprising a central executive that governs attentional control, two domain-specific subsystems—the phonological loop for verbal information and the visuospatial sketchpad for visual and spatial information—and an episodic buffer that integrates information from different domains and links it to long-term memory [3,4]. Working memory has been extensively studied, with research exploring its underlying mechanisms, its critical role in interacting with emerging technologies, and its trainability, as summarized in numerous reviews [[5], [6], [7], [8]]. Given our fundamental understanding of the significance of working memory in information processing [9], it is plausible to believe that working memory might play a critical role in understanding individual differences in interactions with XAI systems, e.g., in facilitating the comprehension of explanations provided by the AI system, integrating new information with existing knowledge, or formulating informed decisions [10]. Findings on cognitive load and the required adjustments to mental models linked with AI explanations further emphasize the importance of working memory in interacting with XAI systems [[11], [12], [13]]. They suggest that individuals with higher working memory capacity derive greater benefits from the explanations provided by the system. Within the 4-D Multiple Resource Model, Wickens [13] points to the crucial role of working memory to managing cognitive load by allocating and integrating cognitive resources across the following four dimensions: processing stages, perceptual modalities, visual channels, and processing codes. Combining the insights from Wickens' model with empirical studies on the impact of different explanations in AI systems on cognitive load [11,12], we propose that working memory plays a critical role in effectively processing and utilizing AI explanations.

Evidence from studies on automation technologies indicate that working memory is related to humans’ performance, and this is independent of the degree of automation [[14], [15], [16]] and its impact on trust in automation [16]. Findings show that lower working memory was associated with an increased level of trust in automation. Rovira et al. [16] argued that individuals with lower working memory capacity had to trust the automation more because they would need to rely on the automation more. In the realm of XAI, we apply a similar rationale, positing that effective interaction with XAI systems necessitates a minimum level of working memory capacity. Consequently, individuals with higher working memory capacities are likely to exhibit a heightened demand for explanations from the system. So far, examinations into the impact of working memory in XAI systems have primarily been indirect or adopted from related fields of research. Therefore, our exploratory study is based on our broad understanding of working memory as well as XAI, serving to bridge this critical gap and offer novel insights into their interplay. Based on previous findings, we hypothesize that people with higher working memory capacity have an increased need for explanations in using AI applications (as they can afford to think more about XAI).

Working memory is a significant predictor of intelligence, particularly fluid intelligence, which involves reasoning, problem-solving, and the ability to learn new information [[17], [18], [19]] - abilities especially relevant for interacting with XAI systems. Intellect, as a personality trait referring to an individual's cognitive capacity is another likely candidate that predicts intelligence, typically self-assessed within the context of the Big Five personality traits [20]. While the Big Five personality traits in general are rather unrelated to working memory, small associations between openness/intellect and working memory have been reported. Specifically, intellect, often considered a facet of Openness to Experience in some personality models [21], has been found to correlate with cognitive performance. For instance, Rozgonjuk et al. [22] found that individuals with higher openness to experience, in particular the facet of self-reported Intellect, exhibited slightly better performance on working memory tasks. Intellect could be seen as a self-assessment regarding one's own cognitive capacities, not necessarily mapping onto one's actual abilities. This is the reason why we aimed to investigate relations between objective WM capacity and self-reported intellect in the context of XAI.

The need for explanations has garnered attention across various domains, including precision medicine, autonomous driving, security, and finance [[23], [24], [25], [26]]. In these fields, decision-making often entails inherent risks, whether in matters of personal health or money. However, the proliferation of AI systems has notably expanded into realms where decision-making holds lesser consequence or none at all, such as the social media sector, entertainment/gaming, or leisure activities. Amidst our increasingly complex world, inundated with a constant stream of information, strategic evaluation of this demand becomes imperative to curb the proliferation of extraneous information. Hence, this study aims to shed light on individuals' varying needs for explanations of AI systems across different contexts representing varying degrees of consequences. We assume a lower demand for XAI in fields with lower levels of (personal) relevance. Here, we focus on three specific areas: the health, social media, and insurance domains. The selection is based on their assumed distinct levels of personal investment and criticality, which were hypothesized to influence the need for XAI. Health and insurance scenarios represent high-stakes contexts where decision outcomes bear significant personal or financial consequences, while social media reflects a more everyday, low-stakes use case. These domains were also chosen to encompass a range of decision-making situations that vary in their assumed frequency and familiarity to participants, allowing us to investigate how context influences the demand for XAI explanations.

## 2 Methods

### 2.1 Participants and procedure

Healthy adults aged 18 years or older were eligible to participate in this study. Additionally, prospective participants were required to self-report normal (or corrected-to-normal) vision and hearing, absence of neurological or psychological disorders, and no diagnosed intellectual or language impairments. Recruitment efforts targeted individuals from the University of Duisburg-Essen and leveraged a German panel provided by Bilendi. Data collection was performed through the online survey platform LimeSurvey, where participants completed the questionnaire and accessed the working memory task through an embedded link.

A total of 237 participants (age range 20–83 years) were recruited for the present online study. To reveal outliers, a boxplot analysis was conducted on working memory performance. Outliers were identified as the score was falling outside the boxplot whiskers, determined using the Tukey (1977) formula: {25th-Quantile - [1.5 x (75th-Quantile - 25th-Quantile)] and {75th-Quantile + [1.5 x (75th-Quantile - 25th-Quantile)]}[13]. For the present sample, 22 participants were identified either as outliers or showed missing data. The remaining sample consisted of 129 females and 86 males aged 20–83 years (M = 35.7 years, SD = 17.1).

The study was performed in accordance with the ethical standards laid down in the Declaration of Helsinki and was approved by the local ethics committee at the University of Duisburg-Essen (ID: 2309APLM6577). All participants provided written informed consent before the experiment and were informed that they could end participation at any time without negative consequences. The study was conducted from the beginning of February to the end of April 2024.

### 2.2 Measures

At the beginning of the experiment, we asked participants to provide data regarding their age, gender, and education. Additionally, participants were asked to indicate whether they had experience with AI in any of the following three areas: 1) health, 2) social media, and 3) insurance.

#### 2.2.1 Working memory

A Number-Position-Binding task was used to assess working memory (a modified version as evaluated earlier in Rozgonjuk et al. [22]). The task was applied in an online version operating seamlessly in any common web browser. Participants engage in this task by learning the positions of numbers on a grid in a round-based format. After each round of memorization, they are required to recall the learned information before proceeding to the next round. In each recall round, either a number was displayed, and the participant had to answer in which of the 9 boxes that number was located, or a box was selected, and the participant had to choose from a list of all learned numbers the one that was previously in the selected box. As grid, a 3x3 system was used, so there were always 9 boxes in total. Depending on the round, between 3 and 6 numbers were learned. Within a round, each number had exactly one individual position. During the learning phase, the word "Attention" was first displayed to the participant in their native language, then the screen was cleared for 0.25 s. The position of the number was then displayed for 2 s, the screen cleared again for 0.25 s, the next number displayed, and so on until all numbers had been shown once. This was followed by the query mentioned above. The Number-Position-Binding task was divided into two sets: a training set with 2 rounds and a trial set with 14 rounds. The training set served to assist the participant with feedback of "Correct" or "Incorrect" to solve the tasks. Subsequently, instructions were displayed explaining that the trial set was beginning and that no feedback on correct or incorrect responses would be given. The trial set was completed exactly once by a participant, regardless of their answers. Finally, the working memory task was concluded with a closing screen. Results can range between 0 and 60.

#### 2.2.2 Intellect/imagination

We used the German version of the Mini-IPIP questionnaire subscale to assess Intellect [20,27]. The personality trait was assessed by four items. Participants were prompted to indicate their level of agreement on a five-point Likert scale, ranging from 1 = strongly disagree to 5 = strongly agree. The sub-score is determined by computing the mean value across the four items (before items were recoded, when necessary). As a result, the score ranges between 1 and 5 points. Cronbach's alpha in our sample was acceptable (α = .63).

#### 2.2.3 Need for explanations in AI systems

In order to get an insight into the need for explanations in AI systems in different contexts, we applied three different scenarios falling in the context of XAI in health, social media, and insurance. Participants were confronted with three short stories describing AI systems 1) for detection of skin cancer (health), 2) in the context of personalization of the social media feed (social media), and 3) in the context of pricing of car insurances (insurance; see appendix). The reason for choosing these three scenarios was mainly to allow us to estimate whether the degree of criticality and consequences, presumably with a higher degree of personal relevance implying higher investment and investment, matters. The scenarios were face validated by being reviewed by three ordinary participants for readability and fitness to purpose and for assessing whether the degree of criticality and investment was seen as we planned it to be. Then participants were asked about their need for explanations of the AI in the three scenarios with the following questions rated from 0 (not important at all) to 10 (extremely important):

1) In this scenario, how important is it for you to understand how AI comes to the decision if the analysed skin region is affected by skin cancer or not?
2) In this scenario, how important is it for you to understand how AI makes car insurance rate decisions?
3) In this scenario, how important is it for you to understand how AI comes to the decision of what to display in the social media feed?

### 2.3 Data analysis

Statistical analyses were conducted using the statistical software Jamovi, version 2.3.21.0 (jamovi.org). Bivariate relationships were examined using Spearman's Rho, with age as a controlled variable. Friedman Test, also controlling for age, was employed to assess the need for explanation across three domains: Health, Social Media, and Insurance. This within-subjects factor was measured at three different points. For post-hoc analysis, the Durbin-Conover test was applied, and Kendall's W was reported as a measure of effect size.

## 3 Results

### 3.1 Participant demographics and descriptive statistics

The final sample consisted of *N* = 215 individuals reporting limited experience with XAI. Participants provided information about their age, gender, education, employment status (cf. Table 1).

**Table 1:** Demographics.

Variables	*N* = 215
**Gender (%)**	
Male	86 (40.0 %)
Female	129 (60.0 %)
**Age**	
M (SD)	35.7 (17.1)
Range	20–83 years
**Education (%)**	
Certificate of Compulsory Basic Secondary Education	11 (5.1 %)
General Certificate of Secondary Education	26 (12.1 %)
Advanced Technical College Certificate	20 (9.3 %)
General University Entrance Qualification	142 (66.0 %)
I'm still a student	4 (1.9 %)
Other Education (Bachelor/Master/vocational school-leaving certificate)	12 (5.6 %)
**Reporting to have** e**xperience (%)**	
AI skin screening application	9 (4.19 %)
AI personalized social media	41 (19.07 %)
AI car insurance	6 (2.79 %)

The mean values, standard deviations, and range of the variables used in further analysis are reported in Table 2. Furthermore, the respective intercorrelations are shown in Table 3.

**Table 2:** Descriptive statistics for all variables.

Variable	*M*	*SD*	Range
Intellect	3.66	0.76	2–5
Working Memory	45.33	8.15	23–57
Need for Explanation (Health)	7.31	2.91	0–10
Need for Explanation (Social Media)	6.20	2.85	0–10
Need for Explanation (Insurance)	7.56	2.40	0–10

**Table 3:** Correlation Matrix*.*

*N* = 215	Intellect	Working Memory	Need for Explanation (Health)	Need for Explanation (Social Media)	Need for Explanation (Insurance)
Intellect	–				
Working Memory	.215∗∗	–			
Need for Explanation (Health)	−.012	.013	–		
Need for Explanation (Social Media)	−.003	−.073	.305∗∗∗	–	
Need for Explanation (Insurance)	.137∗	−.008	.454∗∗∗	.398∗∗∗	–

*Note:* ∗∗*p* < .01, ∗∗∗*p* < .001, presented are values of Spearman's Rho (ρ) when controlling for age.

No significant correlations are visible for working memory, while there are significant moderate to high correlations between the needs for explanation in the three considered contexts. The trait variable intellect shows small to moderate correlations with working memory and the need for explanation in the insurance scenario [28], while there was no significant association found between intellect and the need for explanation in the health and social media context. Visual inspection of the data, along with skewness and kurtosis values within the range of −2 to 2, suggested a normal distribution. However, when Shapiro-Wilk tests were conducted, their results (*p* < .001) indicated a violation of the normality assumption [29]. Given the non-parametric nature of our data, we therefore applied Spearman's rank correlation for a more robust analysis.

### 3.2 Friedman-Test

We tested if the need for explanation differs across contexts (health, social media, and insurance), by using a Friedman-Test. The analysis revealed a significant within-subject effect in the need for explanation (*χ*2(2) = 48.2, *p* < .001, *W* = .112). The post-hoc analysis further breaks down the overall within-effect and allows an investigation of the differences between the needs for explanation in the contexts of health (i.e., understanding how the presented technology detects skin cancer), social media (i.e., comprehending the personalization of a social media feed), and insurance (i.e., apprehending the car insurance technology). A significant difference between the contexts of health and social media (*p* < .001) and between insurance and social media (*p* < .001) was detected. There was no significant difference between health and insurance (*p* = .724).

## 4 Discussion

In this study, we examined the relation of working memory capacity and intellect with the need for explanations when using AI in health, social media, and insurance contexts. Furthermore, we assessed whether the need for explanations varied across these three scenarios. We found that individuals reported a greater need for XAI in scenarios where they were more personally invested, such as health and insurance payment, compared to social media (but see for signifiant differences between the scenarios the result section). The descriptively greater need for XAI in health and insurance scenarios underscores the importance of personal investment and perceived risk. We argue that the higher personal and financial implications in these areas likely heighten the perceived risk, driving a greater demand for clear explanations and increased transparency. This finding aligns with early research indicating that individuals seek more information and clearer explanations when they perceive higher levels of risk [30]. In the context of AI, users demand more interpretable models in high-risk situations to better understand and manage potential consequences [31]. Specific support for the relevance of XAI in the healthcare context comes from previous clinical studies, which emphasize the critical nature of the topic but highlight the strong demand for clear and understandable explanations [32, 33]. According to the Elaboration Likelihood Model, individuals process information through two routes: the central route, involving detailed scrutiny, and the peripheral route, relying on superficial cues [34]. In high-stakes situations, such as health and insurance, individuals are more likely to engage in central processing, motivating them to seek thorough explanations from AI systems to make informed decisions. Conversely, social media, perceived as lower stakes, triggers less need for detailed explanations.

Beyond contextual factors, the type and content of the explanations, as well as individual attributes (e.g., domain knowledge) have been extensively discussed in previous studies on XAI [12,35,36]. Our findings perfectly fit into this body of research, although we found no significant relationship between working memory capacity and the need for XAI across the three domains. Self-reported intellect showed only a mild association with the need for XAI in the insurance context. For the latter, we might argue that individuals who perceive themselves as more cognitively capable are slightly more inclined to seek explanations in complex and financially significant areas like insurance, aligning with the self-efficacy theory [37]. Following this line of reasoning and considering previous findings, we would expect to observe a similar pattern in the health context, what we did not [38]. Together with participants’ assessment of a similar need of explanations in both the health and insurance context, this argument lacks support. However, our findings highlight that while there is a recognized need for XAI across different contexts, the specific attributes influencing this need can vary significantly. This underscores the importance of considering the unique characteristics of each domain when designing and implementing XAI systems.

The significance of our working memory for effectively navigating an increasingly complex world is undeniable, as is its interconnectedness with other cognitive functions [2, 39]. However, studies on working memory training report inconsistent findings regarding their impact on other cognitive functions and the transferability of improvements to everyday-life tasks [40, 41]. Findings are interpreted based on the specificity of tasks used for testing working memory capacity, which may not accurately reflect the complexity and variability of real-life cognitive demands. Furthermore, it is argued that these tasks may not be directly predictive of performance in everyday tasks that require a different set of cognitive skills and strategies. Prior to our study we argued that a certain level of working memory is necessary to understand explanations in XAI. Based on our findings and the relatively high working memory capacity reported within the present sample, we argue that participants generally possessed the working memory capacity needed to understand explanations provided by XAI systems. Therefore, other factors likely contributed to their need for explanations (e.g., technology affinity, adaptability). Their relationship with our working memory in the context of XAI, along with the testing of other cognitive functions, intricately intertwined with working memory, should be the focus of interest in future studies on XAI.

While the present study offers valuable insights, it is imperative to recognize and address its limitations and potential biases. First, our study was conducted through an online survey, which did not involve any direct interaction with an actual XAI system. Instead, we provided participants with information about exemplary/hypothetical applications (health, social media, insurance) and asked them about their need for explanations. This approach has several limitations. The lack of real-world interaction with an XAI system means that participants' responses may not fully capture the complexities and nuances of their actual experiences and needs when using such systems. Additionally, hypothetical scenarios might not elicit the same level of engagement or critical thinking as real-life situations, potentially impacting the accuracy of the findings. Another aspect that needs to be considered, following on from the first aspect of the lack of interaction, concerns the participants' limited previous experience with the XAI applications focused on in this study. Only a small number of people reported that they had any experience with the shown applications (health: n = 9; social media: n = 41; insurance: n = 6). The limited diversity in individual attributes – such as educational background and cognitive capacities – within the current sample represents a constraint, as these factors are known to influence the ability to understand and trust XAI explanations. This limitation may affect the generalizability of the findings, particularly regarding the interaction of different user groups with XAI systems and their interpretation of provided explanations. Therefore, future studies should aim for the recruitment of a more diverse and representative sample. Finally, one might argue that employing a working memory task outside laboratory conditions and embedding it within an online survey introduces various influencing factors beyond our control. While this is a valid concern, past research has demonstrated the reliability of this method for quantification [22]. Additionally, potential individual influencing factors are mitigated by the significantly larger sample sizes compared to traditional laboratory studies. Moreover, we meticulously screened the data for plausibility and reliability. Furthermore, the correlation of demand for XAI with intellect, as well as the significant negative correlation of demand for XAI with age, which we tested during data validation, indicate the robustness of our findings.

## 5 Conclusion/outlook

In conclusion, the present study underscores the imperative for enhanced transparency and detailed explanations in AI systems, particularly within high-stakes domains such as healthcare and insurance, where decision outcomes bear significant personal and financial consequences. Our findings elucidate the complex interplay between individual cognitive attributes, notably self-assessed intellect, and contextual factors that collectively shape the demand for explainability in AI. Notably, while working memory capacity was not found to be a significant predictor of the need for AI explanations across domains, a mild association was observed between self-reported intellect and the demand for explainability in the insurance context. This suggests that users’ self-perceived cognitive competencies may modulate their scrutiny of AI-driven decisions in complex or high-stakes scenarios. Future research is warranted to comprehensively investigate how various individual cognitive traits and contextual parameters influence the demand for explainable AI. Extending this research to encompass a broader spectrum of personality dimensions and cognitive capacities, across diverse application domains, could yield further insights into the conditions under which explainability is deemed essential. Furthermore, a key direction for advancing AI system design lies in developing adaptive frameworks capable of tailoring the granularity and format of explanations to align with user-specific cognitive profiles and the criticality of the domain.

## CRediT authorship contribution statement

**M. Liebherr:** Writing – original draft, Validation, Project administration, Formal analysis, Conceptualization. **E. Gößwein:** Writing – original draft, Formal analysis, Data curation. **C. Kannen:** Writing – original draft, Software, Data curation. **A. Babiker:** Writing – original draft, Conceptualization. **S. Al-Shakhsi:** Writing – original draft, Conceptualization. **V. Staab:** Writing – original draft, Formal analysis. **B.J. Li:** Writing – original draft, Methodology. **R. Ali:** Writing – original draft, Supervision, Conceptualization. **C. Montag:** Writing – original draft, Supervision, Conceptualization.

## Data availability

The data are available upon request from the corresponding author.

## Declaration of competing interest

The authors declare that they have no known competing financial interests or personal relationships that could have appeared to influence the work reported in this paper.
