# Supplementary material for: Working memory and the need for explainable AI – Scenarios from healthcare, social media and insurance
Source: Heliyon. 2025 Jan 10;11(2):e41871. doi: 10.1016/j.heliyon.2025.e41871 (PMC11804545; doi:10.1016/j.heliyon.2025.e41871)
Supplement: Multimedia component 1 [file mmc1.docx]

Appendix

Overview of the Scenarios Encountered by Participants

**Health**

M-Health applications enable users to monitor various aspects of their health on mobile devices. In skin cancer prevention, m-health apps allow users to capture images of skin changes like moles and birthmarks using their mobile devices. AI algorithms can analyze these images to detect patterns that may indicate potential signs of skin cancer. The AI-based diagnosis considers various parameters, including asymmetry, color changes, and texture.

**Social media**

AI in social media personalization leverages user data to create profiles, predict preferences, and curate content. It recommends posts, friends, and ads tailored to individual interests, enhancing user engagement. AI also ranks content, filters spam, and optimizes feed visibility. This personalized experience increases user satisfaction and advertising effectiveness. However, it raises privacy and ethical concerns in data usage and algorithms.

**Insurance**

AI is reshaping car insurance rating by analyzing vast data sets, enabling personalized and fair premium assessments. It employs predictive modeling to identify trends, reducing bias and enhancing transparency. AI also aids in fraud detection and real-time risk monitoring, allowing for dynamic premium adjustments. This might benefit car insurers and policyholders alike by optimizing pricing accuracy and fairness. But it could be also a source of exploitation.
