# Supplementary figures and images for: Working memory and the need for explainable AI – Scenarios from healthcare, social media and insurance
Source: Heliyon. 2025 Jan 10;11(2):e41871. doi: 10.1016/j.heliyon.2025.e41871 (PMC11804545; doi:10.1016/j.heliyon.2025.e41871)

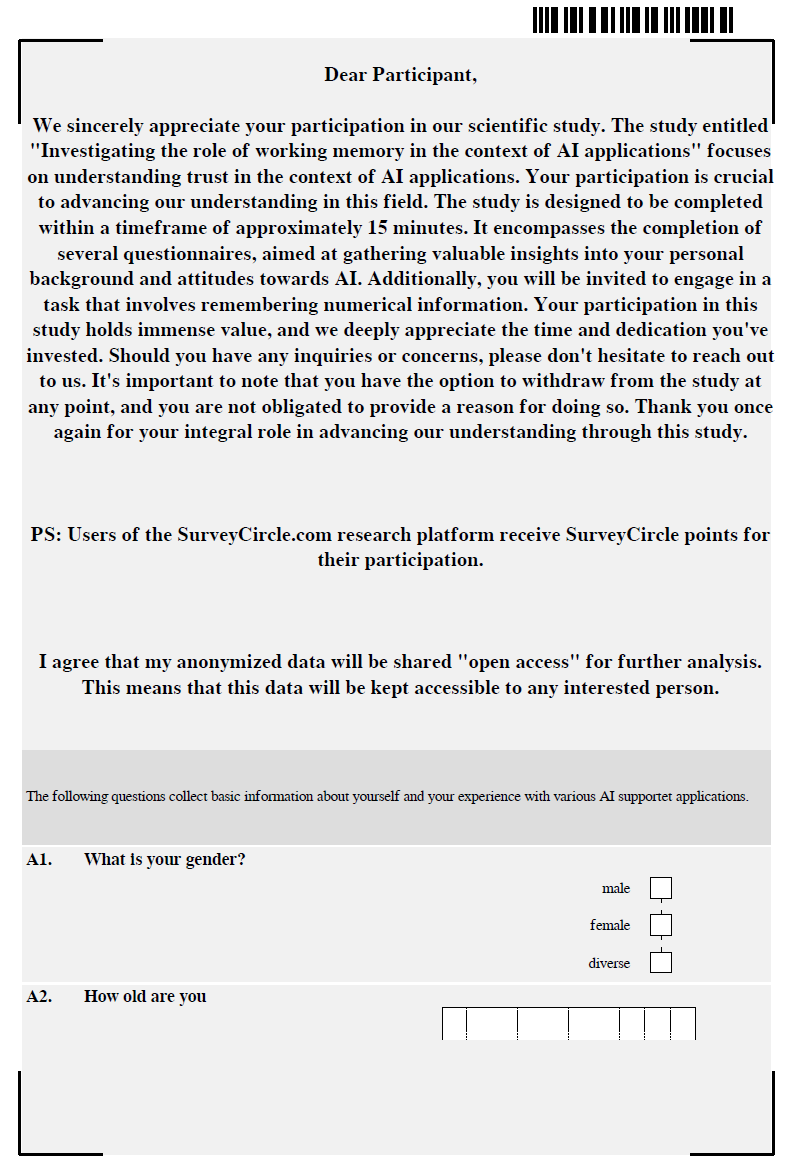


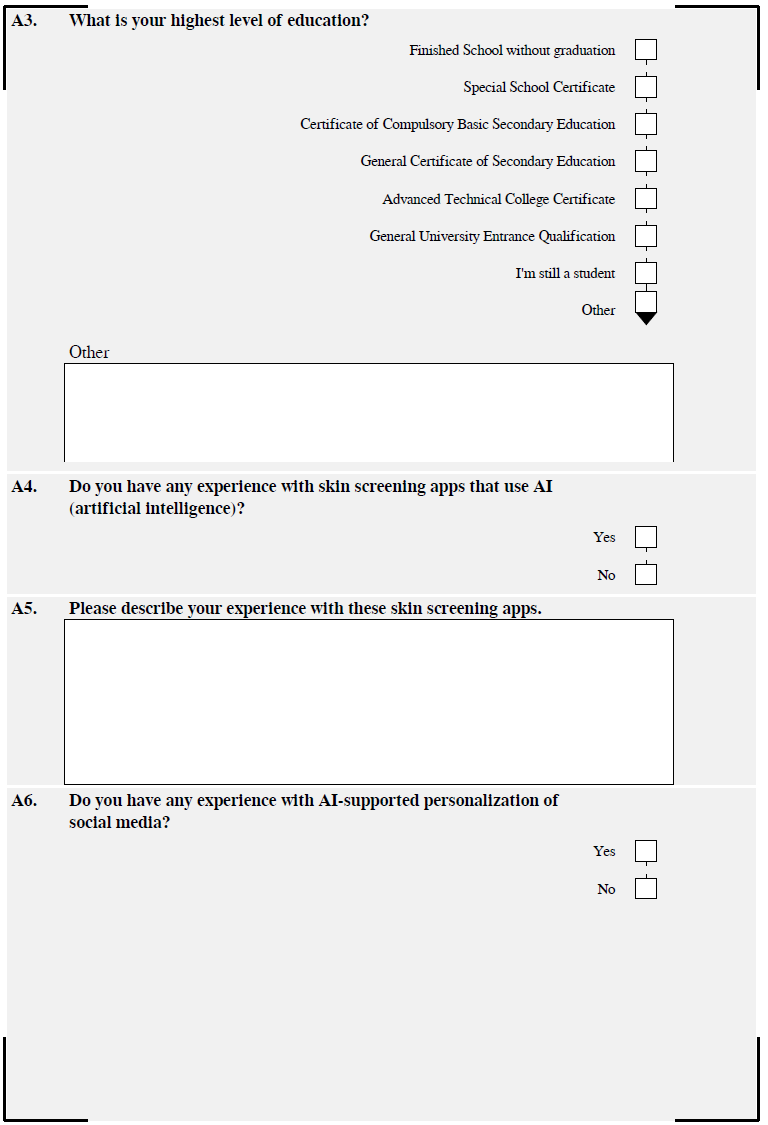


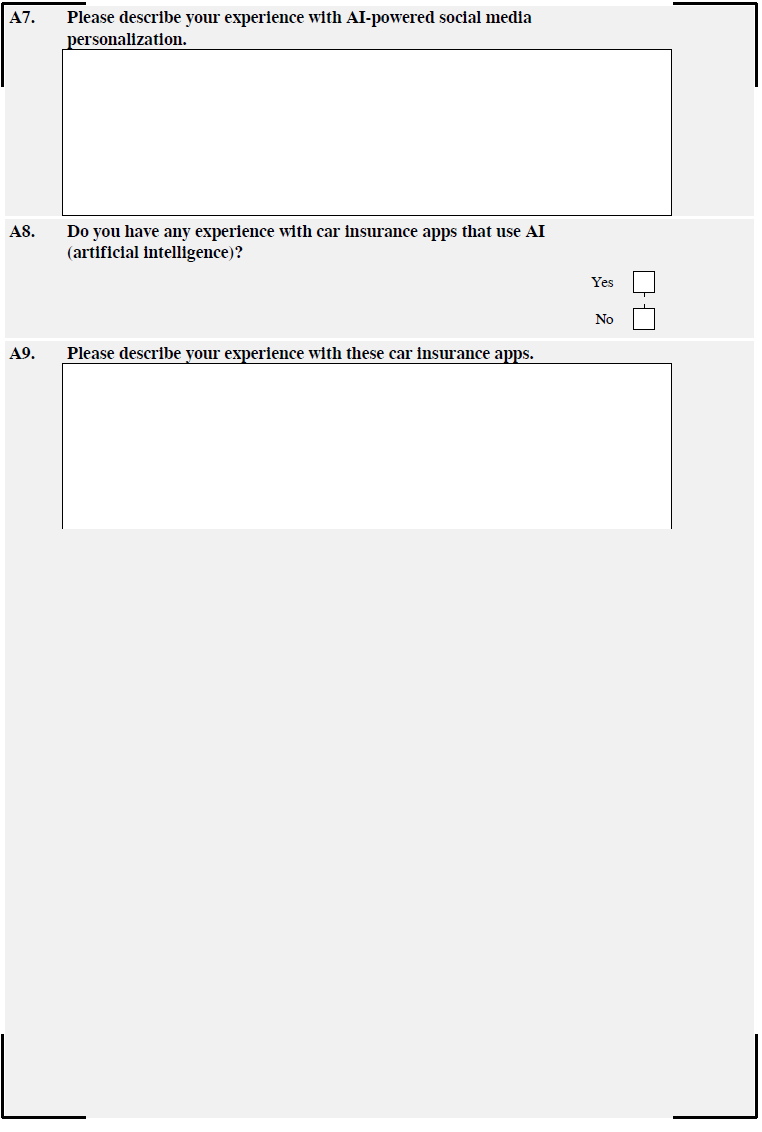


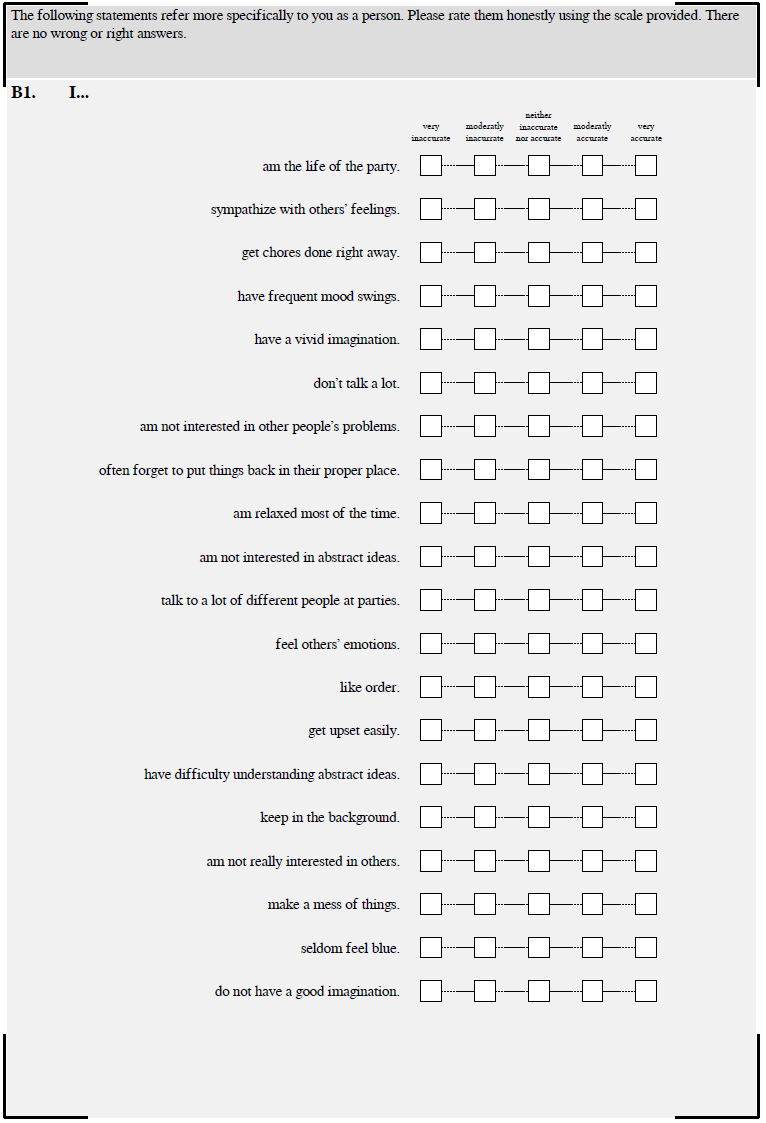


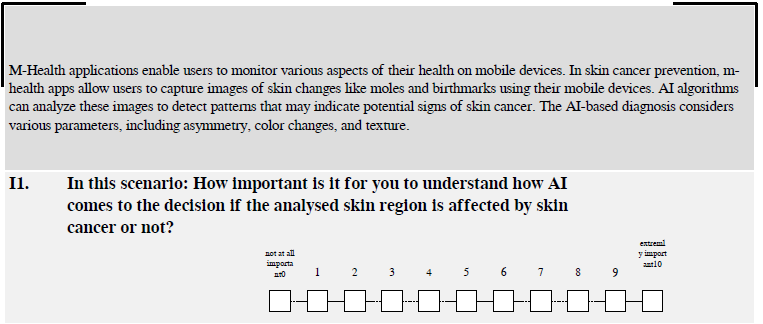


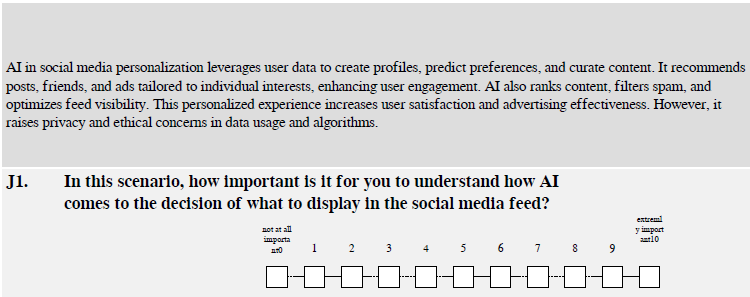


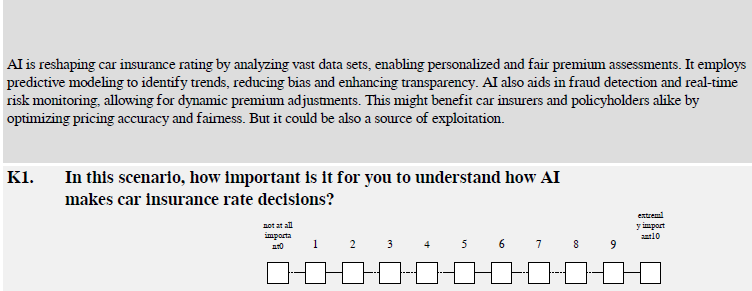

Supplement: Multimedia component 2 [file mmc2.docx]
